# Non-Pleiotropic Coupling of Daily and Seasonal Temporal Isolation in the European Corn Borer

**DOI:** 10.3390/genes9040180

**Published:** 2018-03-26

**Authors:** Rebecca C. Levy, Genevieve M. Kozak, Erik B. Dopman

**Affiliations:** Department of Biology, Tufts University, 200 Boston Ave, Ste. 4700, Medford, MA 02155, USA; reblevy@gmail.com (R.C.L.); genevieve.kozak@tufts.edu (G.M.K.)

**Keywords:** circadian clock, allochronic isolation, gene expression, speciation, sexual behavior

## Abstract

Speciation often involves the coupling of multiple isolating barriers to produce reproductive isolation, but how coupling is generated among different premating barriers is unknown. We measure the degree of coupling between the daily mating time and seasonal mating time between strains of European corn borer (*Ostrinia nubilalis*) and evaluate the hypothesis that the coupling of different forms of allochrony is due to a shared genetic architecture, involving genes with pleiotropic effects on both timing phenotypes. We measure differences in gene expression at peak mating times and compare these genes to previously identified candidates that are associated with changes in seasonal mating time between the corn borer strains. We find that the E strain, which mates earlier in the season, also mates 2.7 h earlier in the night than the Z strain. Earlier daily mating is correlated with the differences in expression of the circadian clock genes cycle, slimb, and vrille. However, different circadian clock genes associate with daily and seasonal timing, suggesting that the coupling of timing traits is maintained by natural selection rather than pleiotropy. Juvenile hormone gene expression was associated with both types of timing, suggesting that circadian genes activate common downstream modules that may impose constraint on future evolution of these traits.

## 1. Introduction

Speciation typically occurs through the accumulation of multiple reproductive barriers [1,2]. The evolution of reproductive isolation is tied to the coupling of different barrier traits that produce increased isolation when acting together when compared to each acting separately [3,4]. Although we understand how coupling between prezygotic and postzygotic isolation occurs through indirect selection during reinforcement [5], the mechanisms that produce coupling between different prezygotic barriers are largely unexplored [3]. It is possible that some prezygotic traits could be coupled through a shared genetic architecture rather than by direct or indirect selection. For example, coupling could occur due to pleiotropy, with a change in a single locus producing multiple types of isolation [6]. Coupling may also emerge from physical linkage or reduced recombination on a chromosome. Such genetic architecture would lead to the rapid evolution of prezygotic reproductive isolation, but it may increase evolutionary constraint of barrier traits and potentially slow the speed of future evolution [7,8,9,10]. In contrast, different and unlinked genes underlying coupled barrier traits could allow for faster, independent evolution of traits, but require selection to generate and maintain coupling of reproductive isolation. Information on pleiotropy’s role in coupling is also needed to estimate the effect on reproductive isolation for any single “speciation” gene [11]. Thus, genetic mechanisms contributing to coupling among barrier traits will have important consequences for the speed, stability, and direction of the evolution of reproductive isolation.

Variation in the timing of mating often contributes to prezygotic reproductive isolation between sympatric populations by reducing the encounter rates between potential mates (eels [12]; salmon [13]; corals [14,15]; mosquitos [16]; moths [17,18,19,20]). Daily (24 h) and seasonal (time of year) forms of allochrony can be coincident and can both limit gene flow between incipient species [21,22], but it is unknown how they become coupled. Given that both forms of allochrony involve endogenous time-keeping, it is possible they rely on the same circadian clock pathway genes [23]. However, there have been few direct comparisons between the genes that are associated with different forms of allochrony in the same species. Studying the mechanisms that produce daily timing variation in a species with seasonal timing variation would allow us to determine if genetic mechanisms underlie coupling, improving our understanding of how these allochronic isolating barriers evolve and lead to speciation [22].

**Figure 1:**
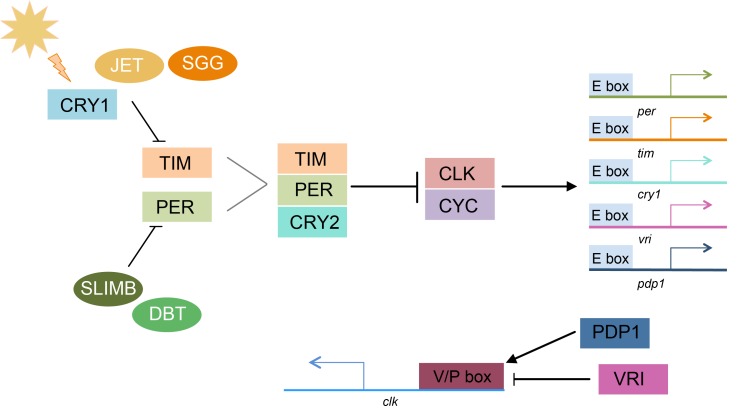
Simplified pathway of the core Lepidopteran circadian clock. Arrows represent activation. Vertical lines represent inhibition. Gray lines indicate dimer formation. During the day, light activates the blue light photoreceptor CRYPTOCHROME1 (CRY1). CRY1 activation leads to SHAGGY (SGG) tagging TIMELESS (TIM) for degradation by JETLAG (JET). The PERIOD (PER) monomer is tagged for degradation by SLIMB when not bound to TIM. DOUBLETIME (DBT) regulates PER activity through phosphorylation. The PER/TIM heterodimer forms a complex with CRYPTOCHROME2 (CRY2) and enters the nucleus, where PER and CRY2 inhibit the activity of the CLOCK/CYCLE heterodimer. CLK/CYC binds to E-box enhancers to activate transcription. A second feedback loop is comprised of VRILLE (VRI) and PDP1, which bind to the V/P box of the *clk* promoter cyclically. VRI acts as repressor of *clk* transcription but PDP1 activates *clk* transcription. Modified from [29].

Variation in daily mating time is the result of variation in the timing of release of sexual signals to attract mates, receptivity to these signals, and the release of gametes [24]. The timing of these traits may often be controlled by the circadian clock pathway, which regulates sexual traits, such as courtship song [25] and pheromone release [24]. The circadian clock synchronizes biological rhythms with 24-h cycles of light and dark using light-sensitive proteins and negative feedback loops (Figure 1) [26]. The heterodimer formed by CLOCK (CLK) and CYCLE (CYC) activates the transcription of a number of circadian genes by binding to E-box sequences in their promoters; these include period (*per*), timeless (*tim*), vrille (*vri*) and PAR domain protein 1 (*pdp1*) [27,28,29,30]. These activated genes feedback to regulate *clk* and *cyc* transcription (Figure 1). Mutations in these core circadian genes have been associated with changes in daily timing of mating, particularly in insects (*Drosophila* [31,32], *Bactrocera* [33,34], *Spodoptera* [35], and *Clunio* [36]).

Although evidence suggests that the differences in daily timing are often associated with the changes in regulation or expression of circadian clock genes, whether daily and seasonal biological rhythms both rely on the circadian clock pathway remains a subject of debate. Seasonal cues, such as increases in temperature and day length (photoperiod), strongly influence the time of year at which insect mating occurs by triggering dormancy break and reproductive development [37]. The light-sensitive circadian clock pathway could potentially sense changes in photoperiod and pleiotropically cause both circadian and seasonal variation in mating time [38]. Diapause response often shows circadian rhythmicity [39] and circadian clock genes do alter the photoperiodic responses that are associated with seasonal timing in species, such as bean bugs (*Riptortus pedestris*), where cycle RNAi impedes diapause break and period RNAi affects diapause entry [40]. However, a functioning endogenous clock does not seem to be necessary for diapause in *Drosophila* [6]. Photoperiodic response can evolve separately from circadian rhythm in pitcher plant mosquitos *Wyeomyia smithii* [41,42], providing further evidence that seasonal timing may be independent of the circadian clock.

Natural variation in daily and seasonal mating time occurs in the European corn borer moth (ECB; *Ostrinia nubilalis*) and causes incomplete reproductive isolation, providing an ideal system to explore coupling and the genetic basis of these traits [4]. Two ECB strains are defined by the use of different pheromones that females use to attract males. In the Z strain, females produce and males preferentially respond to a 3:97 ratio of E and Z isomers of 11-tetradecenyl acetate, while the E strain produces and responds to a 99:1 pheromone ratio [43]. In some locations in North America, sympatric E and Z strains differ in seasonal mating time, with the E strain having a mating flight in May and the Z strain mating flight occurring in July [4,44]. Seasonal timing alone provides incomplete reproductive isolation, limiting 66% of possible matings between strains [4]. Daily temporal isolation has also been reported, but it is unknown if this is coupled with seasonal timing. Using populations with unknown seasonal mating times, Liebherr & Roelofs (1975) observed a two-hour difference in the mean mating time during the night between strains, with Z strain from Ontario mating two hours earlier than E strain from New York [45]. A reversed pattern has been documented in European populations; E-strain females release pheromone and mate earlier than Z strain females [46].

In ECB, seasonal timing is at least partially determined by the time that is needed for overwintering larvae to terminate winter diapause (dormancy) in the spring and summer months [47]. Diapause termination is triggered by a combination of photoperiodic and temperature cues [48] and can be fast (~14 days after receiving diapause breaking cues) or slow (~44 days). These time windows ultimately determine the time of mating flight and the number of generations produced per summer [44,47,49,50]. In New York, E strain moths exhibit fast diapause termination while sympatric Z strain individuals have slow termination [4,44,47]. *Per* is physically linked to the major quantitative trait locus (QTL) controlling the termination time [51,52,53]. Wadsworth & Dopman (2015) quantified gene expression prior to and during diapause termination [54]. Genes that were differentially expressed between strains during diapause termination and/or differing in amino acid sequence included circadian clock genes (*pdp1* and *per*) and several insulin-signaling genes [54]. 

In this study, we investigate differences in the daily mating time between E and Z ECB strains that have been previously characterized for differences in seasonal timing. Strains had been maintained in a common lab environment for several generations to ensure that observed differences among strains are genetic rather than environmentally induced. We test if behavioral nighttime differences in mating are phenotypically coupled to differences in seasonal timing and estimate how much reproductive isolation these differences cause. If *cis*-acting genetic changes in gene regulation underlie differences in mating time, we would expect causal loci to differ in the expression at the peak mating time for each strain. We therefore examine patterns of gene expression associated with changes in times of peak mating activity to identify genetic and regulatory factors that are associated with and potentially contribute to divergence in circadian mating time in ECB. Furthermore, we compare the overlap between genes differentially expressed during mating to candidate genes for seasonal timing (those differentially expressed during diapause termination [54]) to explore the hypothesis that genetic mechanisms produce coupling and that common molecular changes explain the variation in both types of reproductive timing. 

## 2. Methods

### 2.1. Mating Trials

The stocks of bivoltine E (BE) and univoltine Z (UZ) strain ECB were donated by Charles Linn at the New York State Agricultural Experiment Station in Geneva, NY. The BE stock was originally derived from bivoltine E strain individuals collected from Geneva, NY (42.8680° N, 76.9856° W) and the UZ stock from univoltine Z strain individuals that were collected from Bouckville, NY (42.8892° N, 75.5513° W). The stocks were maintained via mass rearing (>200 individuals per generation) at Tufts University for several generations and were the same lines that were previously characterized for seasonal timing [54]. ECB larvae were reared on corn borer diet (Southland Products, Lake Village, Arkansas, USA) under 16:8 L:D, 26 °C conditions until pupation. 300 pupae per strain were removed from rearing containers and placed in individual 44.4 mL plastic cups with lids, each with a 3.8 cm dental wick soaked in water, and returned to the incubator. We replicated methods that were used in a historical study of ECB mating time [45]. One day after eclosion, two males and one female of the same strain were randomly chosen and transferred from individual 44.4 mL cups to one 355 mL transparent plastic container for mating observations. Transfers were completed within the last hour of photophase. During scotophase (16:00–23:00), all of the mating groups were checked for mating activity every 20 min. Each mating group was used only once, with initiation of mating recorded over a 7-h period, for a total of 76 E-strain mating trials and 79 Z-strain trials.

We calculated the absolute strength of the reproductive isolation stemming from mating time following Dopman et al. [4]. We generated the expected number of hybrid and pure-strain offspring after one generation of random mating using Hardy-Weinberg proportions. For each one-hour window (*i*, from 1 to *w*), the expected frequency of hybrid (2*p_i_q_i_*) and pure-strain offspring (*p_i_*^2^ *+ q_i_*^2^) offspring was calculated based on the number of mating moths that were Z strain (*p_i_*) or E strain (*q_i_*). The expected number of hybrid and pure-strain moths each hour was then calculated by multiplying the total number of moths (*n_i_*) by their expected frequencies. Summing the expected numbers of intra- and inter-strain matings across hours (*w*) provided an estimate of the total number of hybrid and parental offspring produced during scotophase. Thus, the strength of reproductive isolation ranges from 0 (random mating) to 1 (complete reproductive isolation), and it is given by Equation (1) below:

(1) $$1-\frac{\sum_{i=1}^{w}\left(2p_{i}q_{i}\right)n_{i}}{\sum_{i=1}^{w}(p_{i}^{2}+q_{i}^{2})n_{i}}$$

### 2.2. Sampling Time Points

In order to identify changes in expression related to circadian control of female mating receptivity, independent of male presence or the physical act of mating, a second experiment was conducted to examine RNA expression levels for the two strains. Additional individuals were reared, as described above, but because mating time is predicted to be female-controlled, individuals were sexed as pupae and only female pupae were kept in isolated 44.4 mL cups until eclosion.

One-day-old virgin females were randomly selected for sacrifice at one of three time points. The first time point was one hour before the end of photophase and provided a daylight baseline for expression change during scotophase (photophase time point). The two other time points corresponded to the median observed mating time for each strain, as determined during the mating trial experiment: 1.3 h into scotophase (median mating time E strain) and 4 h into scotophase (median mating time Z strain). At designated sacrifice time points, the containers were placed in triple-layered black plastic bags and were kept at −20 °C for 10 min to sedate the moths. Under dark conditions, female moths were transferred to 1.5 mL microcentrifuge tubes containing 1.0 mL of RNAlater (Qiagen, Hilden, Germany). Moths in RNAlater were stored at −20 °C for the duration of the experiment, before long-term storage at −80 °C. A total of 72 females of each strain were preserved, and 60 of these were ultimately used for sequencing. 

### 2.3. Library Sequencing

Adult moths were decapitated and heads were pooled according to time point and strain. Heads were used due to known circadian gene expression in antennal and neural tissue [55,56,57,58]. Tissue from five combined heads was used, with four replicate pools for each strain and time point, for a total of 24 pools used for RNA extraction. Pooled heads were used to get sufficient RNA for RNA-seq. RNA was extracted from pooled samples using RNeasy kits (Qiagen, Hilden, Germany). RNA samples were quantified with a Nanodrop (Thermo Scientific, Wilmington, DE, USA) and Qubit Broad Range RNA assays (Life Technologies, Carlsbad, CA, USA).

cDNA libraries were prepared from mRNA using the TruSeq Sample Prep Kit v2 Set A (Illumina Inc., San Diego, CA, USA) using 1 mg total RNA, and prepared libraries were quantified using the Qubit High Sensitivity DNA assay. Libraries were quantified a second time on an Agilent Bioanalyzer (Santa Clara, CA, USA). Two replicate libraries for each strain and time point were run on each of the two lanes of an Illumina HiSeq 2500, located at the Tufts University Core Facility for Genomics (Boston, MA, USA) to generate 100 bp single-end reads from 23 libraries. One UZ 1.3 h library failed. Sequences are available in the GenBank Sequence Read Archive (SRA, accession: SRP135924).

### 2.4. Transcriptome Assembly

Single-end Illumina sequencing reads were assessed for quality using the FastQC program (http://www.bioinformatics.babraham.ac.uk/projects/fastqc). Sequences were then trimmed using Trimmomatic version 0.32 to remove adapter sequences, bases with low sequence quality, and any reads that were shorter than 36 base pairs [59]. FastQC reports were generated for each file again to confirm post-trimming quality. Mitochondrial DNA and ribosomal RNA sequences were removed using Bowtie2 version 2.1.0 [60] by aligning against known mtDNA sequences and identical reads were collapsed prior to assembly (but counts retained) using the FastX Toolkit version 0.013 (http://hannonlab.cshl.edu/fastx_toolkit). The transcriptome was assembled *de novo* using Trinity and a k-mer length of 25 from all reads (both strains and all time points) [61]. The longest transcript for each component were retained using custom scripts and the R package SeqinR [62]. This transcriptome is available in Dryad (doi:10.5061/dryad.k7t20).

### 2.5. Differential Expression Analysis

The set of longest transcripts was indexed as a reference transcriptome, and the individual reads were mapped back to this transcriptome using Bowtie2 version 2.1.0 [60]. The resulting SAM file was parsed by individual libraries to generate count data, which were merged and filtered for weakly expressed genes, defined as genes that did not have at least one count per million reads in at least seven libraries (the minimum number of libraries representing the two strains at a given time point). Libraries were normalized for GC content and between-library sequencing depth using the R package EDASeq [63] and input as offsets along with the raw counts into edgeR [64].

**Figure 2:**
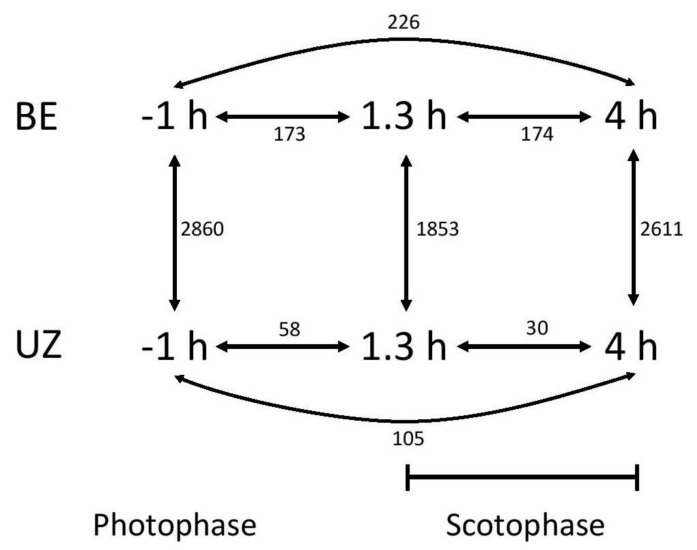
Schematic of specific contrasts tested in differential expression analysis. Within strain, comparisons were made between each pair of time points (photophase vs. 1.3 h of scotophase, photophase vs. 4 h of scotophase, and 1.3 vs. 4 h of scotophase). Between-strain, comparisons were made at each time point (BE vs. UZ). Numbers shown are total numbers of differentially expressed transcripts in each contrast.

Differential expression analysis was done by fitting negative binomial generalized log-linear models in edgeR [64]. Specific contrasts were done between strains at the photophase, 1.3 h, and 4 h time points, as well as within-strain between each pair of time points (Figure 2). *P*-values were corrected for multiple testing using a false-discovery rate (FDR) cutoff of 0.05. Additionally, we emphasized transcripts that were specifically upregulated during at the peak mating time for each strain.

We compared genes that were significantly differentially expressed between strains at any mating time point (photophase, hour 1.3, hour 4; FDR *q* < 0.05) to genes that were differentially expressed between strains during diapause break (day 1 and day 7) from the data set in Wadsworth & Dopman [54]. Orthologous transcripts were identified via blastn and retaining only transcripts with blast scores that were greater than 100.

### 2.6. Annotation

Transcripts were annotated by BLASTing sequences against known *Ostrinia nubilalis*–*Drosophila melanogaster* gene pairs [65]. Supplemental annotations were derived from blasts against the *Bombyx mori* [66] and *Danaus plexippus* proteomes [67]. For circadian transcripts, we list the location in the homologous *B. mori* protein by amino acid number (BMAA). Transcripts were also blasted against known *B. mori* gene pairs to estimate chromosomal locations. Enriched gene ontology (GO) terms were identified for differentially expressed transcripts. This was done using the FlyBase *D. melanogaster* gene IDs [68] derived from the annotated gene pairs and GOrilla [69]. GO enrichment was performed only for transcripts that were also differentially expressed between strains during diapause break. We also searched for previous identified olfactory receptor transcripts from *Ostrinia* using BLASTn searches against previous published mRNA sequences from *Ostrinia furnacalis* [70,71].

Correlations in expression among annotated circadian genes across time points were explored using data from both strains together (six time points total, three E and three Z). For each transcript, normalized read counts were averaged within strain and time point. Correlations in mean expression were calculated across time points and strains using custom scripts and the corrplot package in R [72].

## 3. Results

### 3.1. Mating Trials

**Figure 3:**
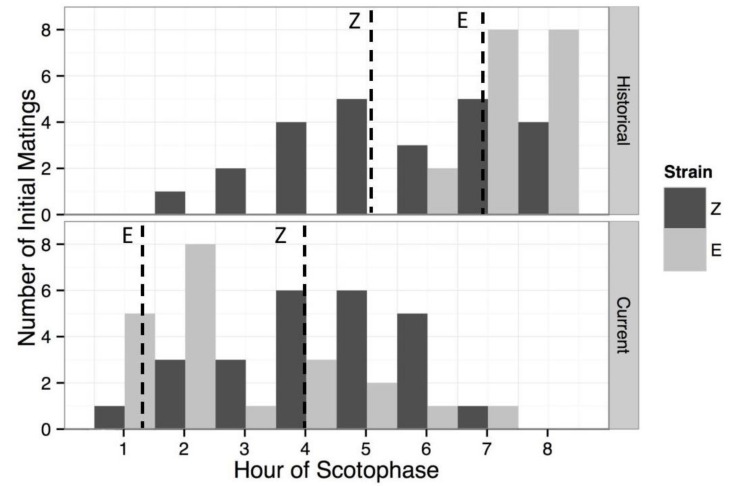
Mating frequencies of the European corn borer E and Z pheromone strains during scotophase. Shown are the number of females mating for the first time during each hour of scotophase, for (**a**) historical field populations with unknown seasonal timing [45] from Aurora, NY (E) and London, Ontario (Z), and (**b**) contemporary colony populations: bivoltine E (Geneva, NY, first mating flight in May), univoltine Z (Bouckville, NY, first mating flight in July). Dashed vertical lines represent median mating times in contemporary populations (E = 1.3, Z = 4.0), and mean mating times for historical populations (E = 6.8, Z = 5.1). Contemporary populations show a significant difference in mean mating time between strains (*p* = 0.0038). When mating times are grouped into 1-h intervals, both of the strains showed significant differences between historical and contemporary mean mating times (E: historical = 7.33, contemporary = 2.81, *p* < 0.0001; Z: historical = 5.58, contemporary = 4.28, *p* = 0.014).

For E strain, a total of 21 of the 76 females (27.6%) mated at any point during the scotophase period. For the Z strain, 25 of the 79 females (31.6%) were observed to mate at any time during the night. The proportions of females mating from each strain were not significantly different (Fisher’s exact test, *p* = 0.602). The median mating times for each strain were 1.33 h into scotophase for the E (mean = 2.41 h) and 4 h for the Z (mean = 3.96). An independent two-group Mann-Whitney-Wilcoxon test indicated that the distributions of the two strains were also significantly different (W = 395, *p* = 0.0035, Figure 3). When mating times were compared with Liebherr & Roelofs [45], Mann-Whitney-Wilcoxon tests showed that the current distribution for the E strain differed from the historical distribution (W = 371, *p* < 0.0001), as did the Z strain distribution (W = 420.5, *p* = 0.014). The absolute strength of circadian mating time as a reproductive isolating barrier between the current E and Z strain populations was calculated to be 0.393, whereas the historical absolute strength of circadian mating time was 0.525.

### 3.2. Transcriptome Assembly

After trimming of raw Illumina reads and removal of mtDNA and rRNA sequences, 61.5 million unique reads remained, representing a total of 78,236 transcripts and 40,811 components. The mean read length was 1136 base pairs, with a minimum of 201, a maximum of 17,430, a median of 691, and an N50 length of 1918 (Table S1).

### 3.3. Differential Expression Analysis

Of transcripts for which differential expression was calculated, a total of 4449 unique transcripts (24.25%) showed patterns of significant differential expression in any contrast. The number of differentially expressed (DE) transcripts varied greatly between the different contrasts tested (Figure 2). Generally, far more DE transcripts were found in between-strain comparisons than any within-strain comparisons.

**Figure 4:**
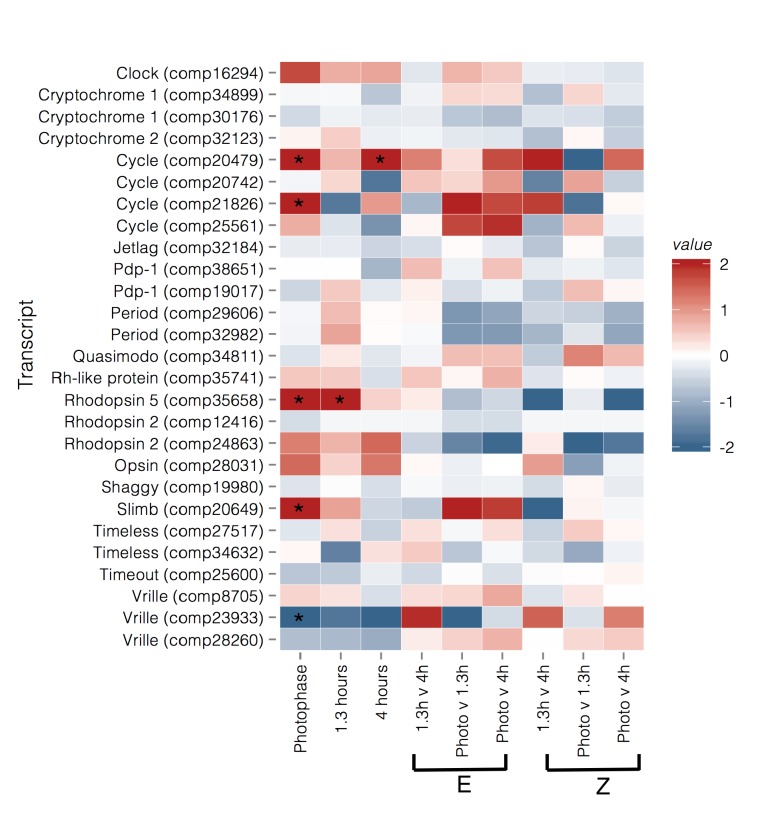
Heatmap of relative expression of genes in the circadian pathway and related photoreceptors in each contrast. Values are log FC. Photophase, 1.3 h, and 4 h contrasts are between strain with red indicating upregulation in BE (early daily and seasonal mating), and blue indicating upregulation in UZ (late daily and seasonal mating). For within strain/between time point contrasts the red color indicates upregulation at the first time point, and blue color indicates upregulation at the second time point. Log FC were capped to a minimum of −2 and a maximum of 2 for visualization purposes. Asterisks indicate genes that are significantly differentially expressed (*q*-value < 0.05) for a given contrast.

**Figure 5:**
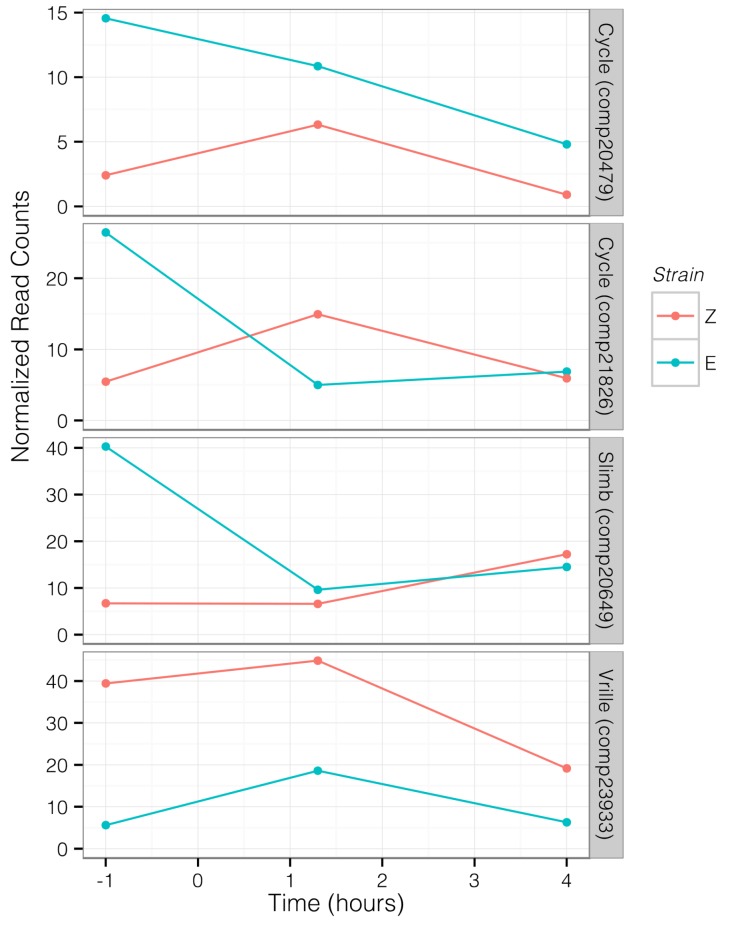
Trajectory of circadian transcript expression levels through time for transcripts that were significantly differentially expressed between strains. Each line represents a single component, with normalized counts at each time point averaged across libraries. Data collection time points were 1 h before scotophase, 1.3 h into scotophase, and 4 h into scotophase. Red indicates values for UZ, blue indicates BE.

Analysis of relative expression levels of 28 transcripts, annotated as circadian or photoreceptive genes, primarily identified transcripts that were differentially expressed between strains during photophase, at E-strain peak mating (hour 1.3), and at Z-strain peak mating (hour 4) (Figure 4 and Figure 5). Two transcripts homologous to *cycle* (*cyc*) were upregulated in the E strain during photophase (comp20479, BMAA 120–180, log_2_ fold change (log FC) = 2.691, *p* = 0.001, *q* = 0.012; comp21826, BMAA 218–294, log FC = 1.96, *p* = 0.003, *q* = 0.02) and 4 h (comp20479, log FC = 2.521, *p* = 0.007, *q* = 0.049). Supernumerary limbs (*slimb*, comp20649, BMAA 144–485) was upregulated in the E strain during photophase (log FC = 2.468, *p* = 2 × 10^−5^, *q* = 0.0003). Vrille (*vri*) was upregulated in the Z strain relative to the E strain during photophase (comp23933, BMAA 227–317, log FC = −2.86, *p* = 0.001, *q* = 0.016, Figure 4 and Figure 5). Clock (*clk*) showed a pattern of higher expression in E during photophase, but this was not significant after correcting for multiple testing (comp16294, BMAA 11–361, log FC = 1.4, *p* = 0.015, *q* = 0.083). Two period (*per*) transcripts were identified but although expression was slightly higher in the E strain at the 1.3 h time point, this was not significant (comp32982, BMAA 732–1108, log FC = 0.74, *p* = 0.07, *q* = 0.28; comp29606, BMAA 1–71, log FC = 0.57, *p* = 0.15, *q* = 0.44). The photoreceptor rhodopsin 5 (*Rh5*, comp35658) was also upregulated in the E strain during photophase and at 1.3 h (log FC = 1.76 and 2.18, respectively). 

We identified transcripts of endocrine system genes downstream of the circadian clock and potentially influencing mating time through the activation of the Pheromone Biosynthesis Activating Neuropeptide (PBAN) pathway [73]. These included allatostatins, biogenic amines (melatonin, serotonin, dopamine, and octopamine), insect hormones (juvenile hormone and ecdysteroid binding proteins), and PBAN. 45 candidate transcripts were identified, of which 19 were significantly differentially expressed in at least one contrast (Figure S1). Six transcripts of takeout juvenile hormone binding proteins showed patterns of differential expression between strains. One transcript (comp30593) was also upregulated within the E strain at hour 1.3 relative to photophase (log FC = 1.344, *p* = 1.59 × 10^−5^, *q* = 0.005). Two genes that were involved in melatonin and ecdysone release in the prothoracic gland also showed differences between strains: Arylalkamine *N*-acetyltransferase (*aaNAT*) was upregulated in the E strain at both photophase (comp26883, log FC = 1.66, *p* = 0.0002, *q* = 0.002) and hour 4 time points (log FC = 1.4, *p* = 0.002, *q* = 0.02); prothoracicotropic hormone (*PTTH*, comp22961) was upregulated in the E strain during photophase (log FC = 1.066, *p* = 0.007, *q* = 0.045). 

Sex peptide receptor is related to the termination of pheromone calling and receptivity to mating [74]. Five transcripts of sex peptide receptor were differentially expressed between strains; four of which were upregulated in the Z strain in at least one contrast. Two transcripts of ecdysis-triggering hormone receptor (comp36183) were upregulated in the E strain during scotophase (transcript a: log FC 4 h = 1.363; transcript b: log FC 1.3 h = 1.023, 4 h = 1.370), while an ecdysone receptor (comp16498, log FC 1.3 h= 1.829, 4 h = 1.583) and ecdysteroid-regulated protein (comp28452, log FC 1.3 h = 1.167; 4 h = 1.265) were upregulated Z strain throughout scotophase (Figure S1). 

Thirteen olfactory receptors (ORs) previously identified in *Ostrinia* were detected in our female head/antennal transcriptome (ORs numbered following [71]). Of these, five were differentially expressed between strains at one or more time points. OR15 was more highly expressed in E strain at photophase (comp28365, log FC = 1.43) and hour 4 (log FC = 1.27). OR37 (comp15171) was more highly expressed in Z strain at photophase (log FC = −8.59) and hour 4 (log FC = −2.91). OR2 (the olfactory co-receptor, comp34293) was more highly expressed in E strain at hour 4 (log FC = 1.17). OR38 was more highly expressed in Z strain at photophase (log FC = −1.80). OR19 was more highly expressed in the Z strain at hour 1.3 (comp12551, log FC = −2.06).

Finally, we identified ten transcripts that fit a predicted pattern of significant differential expression between the strains at both hour 1.3 and hour 4. Of these, six were successfully annotated, including four with immunological functions: two transcripts of cecropin A, and two of attacin. The remaining annotated transcripts were identified as an IQ motif protein and a prickle-like protein.

**Figure 6:**
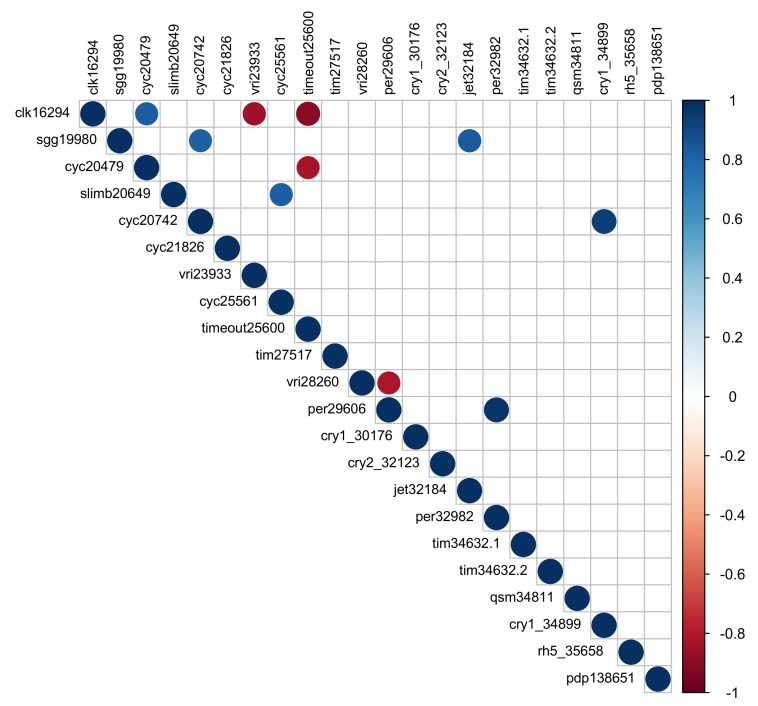
Pairwise correlations among 22 transcripts for circadian genes. Only significant (*p* < 0.05) correlations shown. Blue indicates positive correlations, red indicates negative. Transcripts listed by gene symbol and comp number.

Correlation analysis among 22 circadian clock transcripts identified four transcripts that were significantly negatively correlated across time points and six that were significantly positively correlated with one another (*p* < 0.05; Figure 6). *Clk* (comp16924, BMAA 11–361) was positively correlated with one transcript of *cyc* (comp20479, BMAA 120–180, *r* = 0.82, *p* = 0.044) and negatively correlated with *vri* (comp23933, BMAA 227–317, *r* = −0.84, *p* = 0.036) and timeout (timeless2; comp25600, BMAA 608–1241, r = 0.91, *p* = 0.01). One transcript of *per* (comp29606, BMAA 1–712) was negatively correlated with *vri* (comp28260, BMAA 35–179, *r* = −0.82, *p* = 0.046) and positively correlated with the second transcript of *per* (comp32982, BMAA 732–1108, *r* = 0.97, *p* = 0.001). A second transcript of *cyc* (comp20742, BMAA 494–584) was positively correlated with *cry1* (comp34899, BMAA 1–423), shaggy (*sgg*, BMAA 1–337)*,* and jetlag (*jet*, BMAA 12–217). A third transcript of *cyc* (comp25561, BMAA 694–714) was positively correlated with *slimb* (comp20649, BMAA 144–485, *r* = 0.82, *p* = 0.042).

Transcripts differentially expressed between strains within the daily time course were compared to those that were differentially expressed between the same strains during a diapause termination time course (published data in [54]). We found 1231 transcripts were outliers for at least one time point in both studies. No core circadian clock genes were present among these joint outliers. Four takeout transcripts were differentially expressed in both data sets. We also found another hormonal gene, ecdysis triggering hormone receptor, was an outlier in both data sets. Wadsworth & Dopman [54] identified 41 genes that were differentially expressed during diapause break and physically located near the major QTL for seasonal timing. Six of these genes were differentially expressed at one or two time points in our current study (photophase: tetratricopeptide repeat protein 39B; 1.3 h: rings lost; 4 h: *CG30427*, *magu*; two time points: scabrous, *CG10338*) and three were differentially expressed at all three time points (Table S2). However, none of these genes had been identified in Wadsworth & Dopman as strong candidates for seasonal timing loci based on their function [54].

A total of 6,430 transcripts (15.75%) were annotated with *D. melanogaster* gene IDs. In the between-strain contrasts, enriched GO terms found amongst all of the assembled transcripts that were significantly differentially expressed included catalytic and hydrolytic activity during photophase, structural constituents at hour 1.3, and thiolester hydrolytic activity and the regulation of triglyceride metabolism at hour 4. When separated by strain, additional enriched GO terms were identified. In the E strain, light absorption was enriched at hour 1.3 and pigmentation-related terms and melatonin defense response regulation were enriched at hour 4 (Table S3). In the Z strain, the regulation of wound healing was enriched during photophase. 

Fewer enriched GO terms were identified in within-strain contrasts. Within the E strain, enrichment of voltage-gated ion activity was found in transcripts significantly differentially expressed between hour 1.3 and 4. Within the Z strain, the only GO terms enriched between any of the contrasts were microvillus and actin-based cell projection in the photophase vs. hour 4 contrast. We also performed GO enrichment on 157 annotated joint outliers from the diapause time course. This analysis found that only macromolecule depalmitoylation, pyrimidine nucleobase metabolic process, and palmitoyl-(protein) hydrolase activity were significantly enriched.

## 4. Discussion

Our results indicate that differences in daily mating time are coupled with differences in the seasonal timing in ECB. E strain females that break diapause early in the season mated 2.6 h earlier during scotophase than Z strain females that break diapause later in the season. This coupling will enhance the total isolation among these sympatric ECB strains. Dopman et al. (2010) estimated that seasonal timing differences in New York can limit up to 66% of inter-strain mating [4], based on 10 years of trapping data. We estimate daily mating time further reduces inter-strain mating by 39%. Daily timing is a weaker reproductive isolating barrier on its own when compared to seasonal timing. However, cumulative reproductive isolation is enhanced when later acting barriers limit the portion of gene flow remaining after earlier barriers [4]. When daily mating time is coupled with seasonal timing differences, it could limit an additional 39% of the 34% of gene flow not limited by seasonal timing. Together, these two barriers can limit 79% of inter-strain mating and lead to considerable reproductive isolation in ECB populations. 

We found differences in the daily mating time measured among ECB populations in this study and those that were measured in the historical Liebherr & Roelofs study [45]. Our E strain population originated from a site 50 km distant from the historical E site and our Z strain site was 501 km distant from the historical Z site. In our E and Z strain populations, we found a weaker contribution of mating time to reproductive isolation (39%) than in the historical study (52%) [4]. This suggests that the daily timing and the amount of reproductive isolation that it causes may vary geographically or across years in ECB.

Coupling of daily and seasonal mating time could occur due to selection against recombinant genotypes or pleiotropic alleles influencing both traits [3]. We evaluated the hypothesis that genes within the circadian clock contribute to both seasonal and daily timing differences. Several circadian clock genes were significantly upregulated in one strain relative to the other during our nightly mating time course (Figure 4 and Figure 5). The central regulator of the circadian clock is the CLK/CYC heterodimer which binds to E-box enhancers of various circadian genes, increasing their expression [75]. At the end of photophase, we found levels of *cyc*, and to a lesser extent, *clk*, were higher in the E strain. *Vri* represses *clk* and its expression was lower in the E strain at the same time point [76]. In our correlation analysis, we found that *vri* expression was significantly negatively correlated with *clk*, *cyc*, and *per* expression across multiple time points. The expression of *slimb* was positively correlated with *cyc* across time points and *slimb* was also more highly expressed in the E strain during photophase. SLIMB degrades PER, which should result in the decreased inhibition of CYC [77]. This suggests that earlier mating in the E strain may be tied to differences in levels of *cyc*, *clk*, *vri*, and *slimb* at the end of photophase. Further, it suggests that the circadian clock of the E strain may have a shifted phase relative to that of the Z strain, with peaks in the expression of circadian genes occurring earlier in a given 24-h period, a hypothesis that could be tested by comparing patterns of activity and gene expression between strains over the entire 24-h cycle. Thus, changes in expression of these core circadian clock genes during late photophase may be triggering differences in the timing of pheromone biosynthesis and release, leading to differences in mating time.

Work in other species supports *vri* as an interesting candidate gene for shifting the daily timing of mating behavior. Selected early and late *Drosophila* eclosion chronotypes differ in the timing of the peak expression of *vri* by about 2.5 h during early scotophase [78]. Both *vri* and *slimb* are known to be regulated by ecdysone, the insect steroid hormone that plays a key role in reproduction and gamete release [79,80,81]. Finally, the pattern that we document here, of increased *vri* expression at the end of photophase being associated with later mating, was also found using RT-qPCR between corn and rice strains of the fall armyworm (*Spodoptera frugiperda*) that differ in mating time [35]. Hanniger et al. (2017) found that *vri* was on the same chromosome as the QTL for mating time in armyworms, suggesting that it may be a causal factor for changes in mating time [35]. Thus, *vri* and alterations in the expression of *cyc* and *clk* may be a general mechanism of regulating daily allochrony in moths, as corn borers and armyworms are distantly related moth species (*Ostrinia* are in the Pyraloidea superfamily; *Spodoptera* are in Noctuoidea). 

The circadian clock pathway was hypothesized to be a shared genetic pathway between daily and seasonal allochrony. The circadian clock in the brain acts as the pacemaker that synchronizes the body’s endogenous clock [30,82]. We compared genes that were differentially expressed in adult female heads to those differentially expressed in larval heads during diapause break in ECB [54]. We did not find any core circadian clock genes that differed in both time courses. *Per* is a candidate gene for diapause timing in ECB because it shows amino acids changes among E and Z strains and trends toward higher expression in the E strain during diapause break (days 1 and 7) [54]. In addition, *per* alleles are linked to the QTL for diapause timing and oscillate in frequency across latitude with voltinism (generation number) [52,53]. *Pdp1* is also differentially expressed during diapause break [54] and has been associated with diapause timing in several other insects [83], but neither *per* nor *pdp1* were differentially expressed across the first 4 h of the night. Further characterization of the expression of circadian clock genes is needed determine whether this result is robust across 24-h time periods and rule out that differential expression of *per* does not occur later in the night. Future work can also scan for naturally segregating genetic variation in regulatory or coding sequences between ECB strains in these important timing genes, as we would expect causal genes to contain mutations in *cis*-regulatory or protein-coding regions. Thus, while the circadian clock pathway might be involved in both seasonal and circadian timing, different genes within these pathways are associated with different types of timing. 

Although associated circadian loci for daily and seasonal timing may be different, they may interact with common downstream physiological pathways that lead to phenotypic shifts in the biological rhythm. We did find evidence for shared differential expression of genes in the juvenile hormone pathway. Juvenile hormone (JH) and ecdysteroids play key roles in triggering developmental transitions and reproduction [84]. In moths, JH stimulates female pheromone release [85] and is also involved in maintaining winter dormancy [86,87]. Circadian clock proteins directly regulate a number of genes involved in hormone production, including the takeout family of JH binding proteins [88,89,90]. Expression of takeout alters the timing of male courtship behavior in *Drosophila* [25,90]. Six transcripts from the takeout family were significantly differentially expressed between strains at several night time points and four of these were also differentially expressed between strains during diapause break. For example, comp30593 is upregulated both in E strain when compared to Z strain at hour 1.3 (Figure S1) and in E strain as compared to Z on day 7 after diapause break [54]. These results suggest that different changes in the circadian clock pathway activate the same downstream hormonal module to break diapause or induce mating [6]. Such modularity is common among developmental genes [91] and may constrain the evolution of gene expression within modules [92].

Melatonin is also thought to be important in linking circadian rhythms to behavior, possibly by influencing the release of JH or ecdysteroids [84]. *aaNAT* is a gene that is regulated by *clk* and *cyc* and stimulates melatonin production [93,94]. In our data set, one *aaNAT* transcript was upregulated in the E strain (at photophase and hour 4). *aaNAT* is known to stimulate the release of prothoracicotropic hormone (PTTH). We observed that *PTTH* was also upregulated in the E strain during photophase. Expression differences of *aaNAT* and *PTTH* may represent another instance of daily and seasonal timing involving similar downstream pathways, as these genes are involved in seasonal diapause termination in the moth *Antheraea pernyi* [94,95].

We found little evidence for pleiotropy as a cause of coupling between daily and seasonal allochrony in ECB. Evidence for physical linkage among daily and seasonal differentially expressed candidate genes is also minimal. *Per*, *cyc*, and *pdp1* are all located on the sex (Z) chromosome, while *vri* and *slimb* are autosomal [52,96,97]. *Per* is over 40 centiMorgans (cM) away from *pdp1* and *cyc*. This lack of pleiotropy or genetic linkage in ECB is consistent with the work in pitcher plant mosquitoes that found that seasonal and daily timing traits evolve independently under artificial selection in mosquitos [6,41]. While such a genetic architecture suggests that future evolution of different types of allochrony could occur independently in ECB, the common activation of downstream hormone modules creates a susceptibility to pleiotropy and could produce constraints on the future evolution of these traits [8]. 

Given that coupling among different forms of allochronic isolation in ECB is not due to shared or physically linked genes, coupling is likely maintained by selection and occurs because recombinant individuals have low fitness [3]. For example, reproductive output may be reduced in individuals who emerge from diapause in early spring, but reproduce later in the night (or individuals that emerge from diapause in the summer but reproduce early in the night) because they have difficulty finding mates. Future work using recombinant lines to decouple these phenotypes could test these hypothesized fitness effects as well as characterize their genetic architectures. Coupling of timing traits to other forms of reproductive isolation may further strengthen total reproductive isolation among sympatric ECB strains. Sexual isolation among E and Z strains occurs due to female pheromone production and male pheromone response, limiting ~79% of inter-strain mating [4]. Timing loci are not genetically linked to the autosomal pheromone gene (*pgFAR*) and the major QTL for male response on the Z chromosome is far from *per* (11cM) and *pdp1* (21 cM) [51,96,98]. Thus, natural selection against hybrid phenotypes is likely to cause coupling of daily allochrony, seasonal allochrony, and sexual isolation in ECB, leading to almost complete reproductive isolation (95%) in geographic locations where populations differ in all three forms of isolation. 

## Supplementary Materials

The following are available online at http://www.mdpi.com/2073-4425/9/4/180/s1, Figure S1: Heatmap of relative expression of downstream candidate transcripts in each contrast, Table S1: Summary statistics of transcriptome assembly in Trinity, Table S2: Overlapping differentially expressed genes between daily and seasonal time courses, Table S3: Summary of GO term enrichment among significantly upregulated genes within each strain at each time point.

## References

[1] Coyne J.A., Orr H.A. (2004). Speciation.

[2] Sobel J.M., Chen G.F., Watt L.R., Schemske D.W. (2010). The Biology of Speciation. Evolution.

[3] Butlin R.K., Smadja C.M. (2017). Coupling, Reinforcement, and Speciation. Am. Nat..

[4] Dopman E.B., Robbins P.S., Seaman A. (2010). Components of reproductive isolation between North American pheromone strains of the European corn borer. Evolution.

[5] Ortiz-Barrientos D., Grealy A., Nosil P. (2009). The Genetics and Ecology of Reinforcement. Ann. N. Y. Acad. Sci..

[6] Emerson K.J., Bradshaw W.E., Holzapfel C.M. (2009). Complications of complexity: Integrating environmental, genetic and hormonal control of insect diapause. Trends Genet..

[7] Arnold S.J. (1992). Constraints on phenotypic evolution. Am. Nat..

[8] Fraser H.B., Hirsh A.E., Steinmetz L.M., Scharfe C., Feldman M.W. (2002). Evolutionary rate in the protein interaction network. Science.

[9] Barton N.H. (1990). Pleiotropic models of quantitative variation. Genetics.

[10] Griswold C.K., Whitlock M.C. (2003). The Genetics of Adaptation: The Roles of Pleiotropy, Stabilizing Selection and Drift in Shaping the Distribution of Bidirectional Fixed Mutational Effects. Genetics.

[11] Seehausen O., Butlin R.K., Keller I., Wagner C.E., Boughman J.W., Hohenlohe P.A., Peichel C.L., Sætre G.-P., Bank C., Brännström Å. (2014). Genomics and the origin of species. Nat. Rev. Genet..

[12] Maes G.E., Pujolar J.M., Hellemans B., Volckaert F.A.M. (2006). Evidence for isolation by time in the European eel (*Anguilla anguilla* L.). Mol. Ecol..

[13] O’Malley K.G., Camara M.D., Banks M.A. (2007). Candidate loci reveal genetic differentiation between temporally divergent migratory runs of Chinook salmon (*Oncorhynchus tshawytscha*). Mol. Ecol..

[14] Fukami H., Omori M., Shimoike K., Hayashibara T., Hatta M. (2003). Ecological and genetic aspects of reproductive isolation by different spawning times in *Acropora* corals. Mar. Biol..

[15] Levitan D.R., Fukami H., Jara J., Kline D., McGovern T.M., McGhee K.E., Swanson C.A., Knowlton N. (2004). Mechanisms of reproductive isolation among sympatric broadcast-spawning corals of the *Montastraea annularis* species complex. Evolution.

[16] Sawadogo S.P., Costantini C., Pennetier C., Diabaté A., Gibson G., Dabiré R.K. (2013). Differences in timing of mating swarms in sympatric populations of Anopheles coluzzii and *Anopheles gambiae* s.s. (formerly *A. gambiae* M and S molecular forms) in Burkina Faso, West Africa. Parasit. Vectors.

[17] Pashley D.P., Hammond A.M., Hardy T.N. (1992). Reproductive isolating mechanisms in fall armyworm host strains (Lepidoptera: Noctuidae). Ann. Entomol. Soc. Am..

[18] Monti L., Genermont J., Malosse C., Lalanne Cassou B. (1997). A genetic analysis of some components of reproductive isolation between two closely related species, *Spodoptera latifascia* (Walker) and *S. descoinsi* (Lalanne-Cassou and Silvain) (Lepidoptera: Noctuidae). J. Evol. Biol..

[19] Ueno H., Furukawa S., Tsuchida K. (2006). Difference in the time of mating activity between host-associated populations of the rice stem borer, *Chilo suppressalis* (Walker). Entomol. Sci..

[20] Huang C., Hu B., Li J., Wang Y. (2016). Water-oats harbors two strains of the striped stem borer *Chilo suppressalis* (Lepidoptera: Crambidae) with temporal divergence in mating behavior. Appl. Entomol. Zool..

[21] Hendry A.P., Day T. (2005). Population structure attributable to reproductive time: Isolation by time and adaptation by time. Mol. Ecol..

[22] Taylor R.S., Friesen V.L. (2017). The role of allochrony in speciation. Mol. Ecol..

[23] Denlinger D.L., Hahn D.A., Merlin C., Holzapfel C.M., Bradshaw W.E. (2017). Keeping time without a spine: What can the insect clock teach us about seasonal adaptation?. Philos. Trans. R. Soc. B Biol. Sci..

[24] Krupp J.J., Billeter J.-C., Wong A., Choi C., Nitabach M.N., Levine J.D. (2013). Pigment-dispersing factor modulates pheromone production in clock cells that influence mating in *Drosophila*. Neuron.

[25] Dauwalder B., Tsujimoto S., Moss J., Mattox W. (2002). The *Drosophila takeout* gene is regulated by the somatic sex-determination pathway and affects male courtship behavior. Genes Dev..

[26] Zheng X., Sehgal A. (2008). Probing the relative importance of molecular oscillations in the circadian clock. Genetics.

[27] Hardin P.E. (2005). The circadian timekeeping system of *Drosophila*. Curr. Biol..

[28] Emery P., So W.V., Kaneko M., Hall J.C., Rosbash M. (1998). CRY, a *Drosophil*a clock and light-regulated *cryptochrome*, is a major contributor to circadian rhythm resetting and photosensitivity. Cell.

[29] Zhan S., Merlin C., Boore J.L., Reppert S.M. (2011). The monarch butterfly genome yields insights into long-distance migration. Cell.

[30] Sandrelli F., Costa R., Kyriacou C.P., Rosato E. (2008). Comparative analysis of circadian clock genes in insects. Insect Mol. Biol..

[31] Sakai T., Ishida N. (2001). Circadian rhythms of female mating activity governed by clock genes in *Drosophila*. Proc. Natl. Acad. Sci. USA.

[32] Tauber E., Roe H., Costa R., Hennessy J.M., Kyriacou C.P. (2003). Temporal mating isolation driven by a behavioral gene in *Drosophila*. Curr. Biol..

[33] Fuchikawa T., Sanada S., Nishio R., Matsumoto A., Matsuyama T., Yamagishi M., Tomioka K., Tanimura T., Miyatake T. (2010). The clock gene *cryptochrome* of *Bactrocera cucurbitae* (Diptera: Tephritidae) in strains with different mating times. Heredity.

[34] Miyatake T., Matsumoto A., Matsuyama T., Ueda H.R., Toyosato T., Tanimura T. (2002). The period gene and allochronic reproductive isolation in *Bactrocera cucurbitae*. Proc. R. Soc. B Biol. Sci..

[35] Hänniger S., Dumas P., Schöfl G., Gebauer-Jung S., Vogel H., Unbehend M., Heckel D.G., Groot A.T. (2017). Genetic basis of allochronic differentiation in the fall armyworm. BMC Evol. Boil..

[36] Poehn B., Szkiba D., Preussner M., Sedlazeck F.J., Zrim A., Neumann T., Nguyen L.-T., Betancourt A.J., Hummel T., Vogel H. (2016). The genomic basis of circadian and circalunar timing adaptations in a midge. Nature.

[37] Koštál V. (2006). Eco-physiological phases of insect diapause. J. Insect Physiol..

[38] Meuti M.E., Denlinger D.L. (2013). Evolutionary links between circadian clocks and photoperiodic diapause in insects. Integr. Comp. Biol..

[39] Saunders D.S., Lewis R.D., Warman G.R. (2004). Photoperiodic induction of diapause: Opening the black box. Physiol. Entomol..

[40] Ikeno T., Numata H., Goto S.G. (2011). Circadian clock genes *period* and *cycle* regulate photoperiodic diapause in the bean bug *Riptortus pedestris* males. J. Insect Physiol..

[41] Bradshaw W.E., Holzapfel C.M., Mathias D. (2006). Circadian rhythmicity and photoperiodism in the pitcher-plant mosquito: Can the seasonal timer evolve independently of the circadian clock?. Am. Nat..

[42] Bradshaw W.E., Emerson K.J., Holzapfel C.M. (2012). Genetic correlations and the evolution of photoperiodic time measurement within a local population of the pitcher-plant mosquito, *Wyeomyia smithii*. Heredity.

[43] Kochansky J., Cardé R.T., Liebherr J., Roelofs W.L. (1975). Sex pheromone of the European corn borer, *Ostrinia nubilalis* (Lepidoptera: Pyralidae), in New York. J. Chem. Ecol..

[44] Glover T.J., Knodel J.J., Robbins P.S., Eckenrode C.J., Roelofs W.L. (1991). Gene flow among three races of European corn norers (Lepidoptera: Pyralidae) in New York State. Environ. Entomol..

[45] Liebherr J., Roelofs W.L. (1975). Laboratory Hybridization and Mating Period Studies Using Two Pheromones Strains of *Ostrinia nubilalis*. Ann. Entomol. Soc. Am..

[46] Kárpáti Z., Molnar B., Szőcs G. (2007). Pheromone titer and mating frequency of E- and Z-strains of the European corn borer, *Ostrinia nubilalis*: Fluctuation during scotophase and age dependence. Acta Phytopathol. Entomol. Hung..

[47] Glover T.J., Robbins P.S., Eckenrode C.J., Roelofs W.L. (1992). Genetic control of voltinism characteristics in European corn borer races assessed with a marker gene. Arch. Insect Biochem. Physiol..

[48] McLeod D.G.R., Beck S.D. (1963). Photoperiodic termination of diapause in an insect. Biol. Bull..

[49] Dopman E.B., Perez L., Bogdanowicz S.M., Harrison R.G. (2005). Consequences of reproductive barriers for genealogical discordance in the European corn borer. Proc. Natl. Acad. Sci. USA.

[50] Wadsworth C.B., Woods W.A., Hahn D.A., Dopman E.B. (2013). One phase of the dormancy developmental pathway is critical for the evolution of insect seasonality. J. Evol. Biol..

[51] Dopman E.B., Bogdanowicz S.M., Harrison R.G. (2004). Genetic mapping of sexual isolation between E and Z pheromone strains of the European corn norer (*Ostrinia nubilalis*). Genetics.

[52] Levy R.C., Kozak G.M., Wadsworth C.B., Coates B.S., Dopman E.B. (2015). Explaining the sawtooth: Latitudinal periodicity in a circadian gene correlates with shifts in generation number. J. Evol. Biol..

[53] Wadsworth C.B., Li X., Dopman E.B. (2015). A recombination suppressor contributes to ecological speciation in *Ostrinia* moths. Heredity.

[54] Wadsworth C.B., Dopman E.B. (2015). Transcriptome profiling reveals mechanisms for the evolution of insect seasonality. J. Exp. Biol..

[55] Giebultowicz J.M. (2000). Molecular mechanism and cellular distribution of insect circadian clocks. Annu. Rev. Entomol..

[56] Schuckel J., Siwicki K.K., Stengl M. (2007). Putative circadian pacemaker cells in the antenna of the hawkmoth *Manduca sexta*. Cell Tissue Res..

[57] Merlin C., Lucas P., Rochat D., François M.-C., Maïbèche-Coisne M., Jacquin-Joly E. (2007). An antennal circadian clock and circadian rhythms in peripheral pheromone reception in the moth *Spodoptera littoralis*. J. Biol. Rhythm..

[58] Kobelková A., Závodská R., Sauman I., Bazalová O., Doležel D. (2015). Expression of clock genes *period* and *timeless* in the central nervous system of the Mediterranean flour moth, *Ephestia kuehniella*. J. Biol. Rhythm..

[59] Bolger A.M., Lohse M., Usadel B. (2014). Trimmomatic: A flexible trimmer for Illumina sequence data. Bioinformatics.

[60] Langmead B., Salzberg S.L. (2012). Fast gapped-read alignment with Bowtie 2. Nat. Methods.

[61] Grabherr M.G., Haas B.J., Yassour M., Levin J.Z., Thompson D.A., Amit I., Adiconis X., Fan L., Raychowdhury R., Zeng Q. (2011). Full-length transcriptome assembly from RNA-Seq data without a reference genome. Nat. Biotechnol..

[62] Charif D., Lobry J.R. (2007). SeqinR 1.0-2: A contributed package to the R project for statistical computing devoted to biological sequences retrieval and analysis. Structural Approaches to Sequence Evolution.

[63] Risso D., Schwartz K., Sherlock G., Dudoit S. (2011). GC-content normalization for RNA-seq data. BMC Bioinform..

[64] Robinson M.D., McCarthy D.J., Smyth G.K. (2010). edgeR: A Bioconductor package for differential expression analysis of digital gene expression data. Bioinformatics.

[65] Al-Wathiqui N., Lewis S.M., Dopman E.B. (2014). Using RNA sequencing to characterize female reproductive genes between Z and E Strains of European corn borer moth (*Ostrinia nubilalis*). BMC Genom..

[66] Shimomura M., Minami H., Suetsugu Y., Ohyanagi H., Satoh C., Antonio B., Nagamura Y., Kadono-Okuda K., Kajiwara H., Sezutsu H. (2009). KAIKObase: An integrated silkworm genome database and data mining tool. BMC Genom..

[67] Zhan S., Reppert S.M. (2012). MonarchBase: The monarch butterfly genome database. Nucleic Acids Res..

[68] Dos Santos G., Schroeder A.J., Goodman J.L., Strelets V.B., Crosby M.A., Thurmond J., Emmert D.B., Gelbart W.M., Consortium F. (2014). FlyBase: Introduction of the *Drosophila melanogaster* Release 6 reference genome assembly and large-scale migration of genome annotations. Nucleic Acids Res..

[69] Eden E., Navon R., Steinfeld I., Lipson D., Yakhini Z. (2009). GOrilla: A tool for discovery and visualization of enriched GO terms in ranked gene lists. BMC Bioinf..

[70] Zhang T., Coates B.S., Ge X., Bai S., He K., Wang Z. (2015). Male-and female-biased gene expression of olfactory-related genes in the antennae of Asian corn borer, *Ostrinia furnacalis* (Guenée) (Lepidoptera: Crambidae). PLoS ONE.

[71] Yang B., Ozaki K., Ishikawa Y., Matsuo T. (2015). Identification of candidate odorant receptors in Asian corn borer *Ostrinia furnacalis*. PLoS ONE.

[72] Wei T., Simko V. (2016). corrplot Visualization of a Correlation Matrix.

[73] Groot A.T. (2014). Circadian rhythms of sexual activities in moths: A review. Front. Ecol. Evol..

[74] Hanin O., Azrielli A., Zakin V., Applebaum S., Rafaeli A. (2011). Identification and differential expression of a sex-peptide receptor in *Helicoverpa armigera*. Insect Biochem. Mol. Biol..

[75] Hao H., Allen D.L., Hardin P.E. (1997). A circadian enhancer mediates PER-dependent mRNA cycling in *Drosophila melanogaster*. Mol. Cell. Biol..

[76] Glossop N.R., Houl J.H., Zheng H., Ng F.S., Dudek S.M., Hardin P.E. (2003). VRILLE feeds back to control circadian transcription of *Clock* in the *Drosophila* circadian oscillator. Neuron.

[77] Ko H.W., Jiang J., Edery I. (2002). Role for *Slimb* in the degradation of *Drosophila Period* protein phosphorylated by *Doubletime*. Nature.

[78] Nikhil K.L., Abhilash L., Sharma V.K. (2016). Molecular correlates of circadian clocks in fruit fly *Drosophila melanogaster* populations exhibiting early and late emergence chronotypes. J. Biol. Rhythm..

[79] Koolman J. (1994). Control of ecdysone biosynthesis in insects. Neth. J. Zool..

[80] Beckstead R.B., Lam G., Thummel C.S. (2005). The genomic response to 20-hydroxyecdysone at the onset of *Drosophila* metamorphosis. Genome Biol..

[81] Ables E.T., Hwang G.H., Finger D.S., Hinnant T.D., Drummond-Barbosa D. (2016). A genetic mosaic screen reveals ecdysone-responsive genes regulating *Drosophila* oogenesis. G3 Genes Genomes Genet..

[82] Nitabach M.N., Blau J., Holmes T.C. (2002). Electrical silencing of *Drosophila* pacemaker neurons stops the free-running circadian clock. Cell.

[83] Ragland G.J., Keep E. (2017). Comparative transcriptomics support evolutionary convergence of diapause responses across *Insecta*. Physiol. Entomol..

[84] Bloch G., Hazan E., Rafaeli A. (2013). Circadian rhythms and endocrine functions in adult insects. J. Insect Physiol..

[85] Cusson M., Tobe S.S., McNeil J.N. (1994). Juvenile hormones: Their role in the regulation of the pheromonal communication system of the armyworm moth, *Pseudaletia unipuncta*. Arch. Insect Biochem. Physiol..

[86] Yagi S., Fukaya M. (1974). Juvenile hormone as a key factor regulating larval diapause of the rice stem borer, *Chilo suppressalis* (Lepidoptera: Pyralidae). Appl. Entomol. Zool..

[87] Nijhout H.F., Williams C.M. (1974). Control of moulting and metamorphosis in the tobacco hornworm, *Manduca sexta* (L.): Growth of the last-instar larva and the decision to pupate. J. Exp. Biol..

[88] So W.V., Sarov-Blat L., Kotarski C.K., McDonald M.J., Allada R., Rosbash M. (2000). *Takeout*, a novel *Drosophila* gene under circadian clock transcriptional regulation. Mol. Cell. Biol..

[89] Saito K., Su Z.H., Emi A., Mita K., Takeda M., Fujiwara Y. (2006). Cloning and expression analysis of takeout/JHBP family genes of silkworm, *Bombyx mori*. Insect Mol. Biol..

[90] Benito J., Hoxha V., Lama C., Lazareva A.A., Ferveur J.-F., Hardin P.E., Dauwalder B. (2010). The circadian output gene *takeout* is regulated by Pdp1ε. Proc. Natl. Acad. Sci. USA.

[91] Wagner G.P., Pavlicev M., Cheverud J.M. (2007). The road to modularity. Nat. Rev. Genet..

[92] Fraser H.B. (2005). Modularity and evolutionary constraint on proteins. Nat. Genet..

[93] Dai F.-Y., Qiao L., Tong X.-L., Cao C., Chen P., Chen J., Lu C., Xiang Z.-H. (2010). Mutations of an arylalkylamine-*N*-acetyltransferase, Bm-iAANAT, are responsible for silkworm melanism mutant. J. Biol. Chem..

[94] Mohamed A.A., Wang Q., Bembenek J., Ichihara N., Hiragaki S., Suzuki T., Takeda M. (2014). *N*-acetyltransferase (nat) is a critical conjunct of photoperiodism between the circadian system and endocrine axis in *Antheraea pernyi*. PLoS ONE.

[95] Sauman I., Reppert S.M. (1996). Molecular characterization of prothoracicotropic hormone (PTTH) from the giant silkmoth *Antheraea pernyi*: Developmental appearance of PTTH-expressing cells and relationship to circadian clock cells in central brain. Dev. Biol..

[96] Koutroumpa F.A., Groot A.T., Dekker T., Heckel D.G. (2016). Genetic mapping of male pheromone response in the European corn borer identifies candidate genes regulating neurogenesis. Proc. Natl. Acad. Sci. USA.

[97] Kozak G.M., Wadsworth C.B., Kahne S.C., Bogdanowicz S.M., Harrison R.G., Coates B.S., Dopman E.B. (2017). A combination of sexual and ecological divergence contributes to rearrangement spread during initial stages of speciation. Mol. Ecol..

[98] Lassance J.-M., Groot A.T., Liénard M.A., Antony B., Borgwardt C., Andersson F., Hedenström E., Heckel D.G., Lofstedt C. (2010). Allelic variation in a fatty-acyl reductase gene causes divergence in moth sex pheromones. Nature.

