# Supplementary material for: Non-Pleiotropic Coupling of Daily and Seasonal Temporal Isolation in the European Corn Borer
Source: Genes (Basel). 2018 Mar 26;9(4):180. doi: 10.3390/genes9040180 (PMC5924522; doi:10.3390/genes9040180)
Supplement: Supplementary file 1 [file genes-09-00180-s001.pdf]

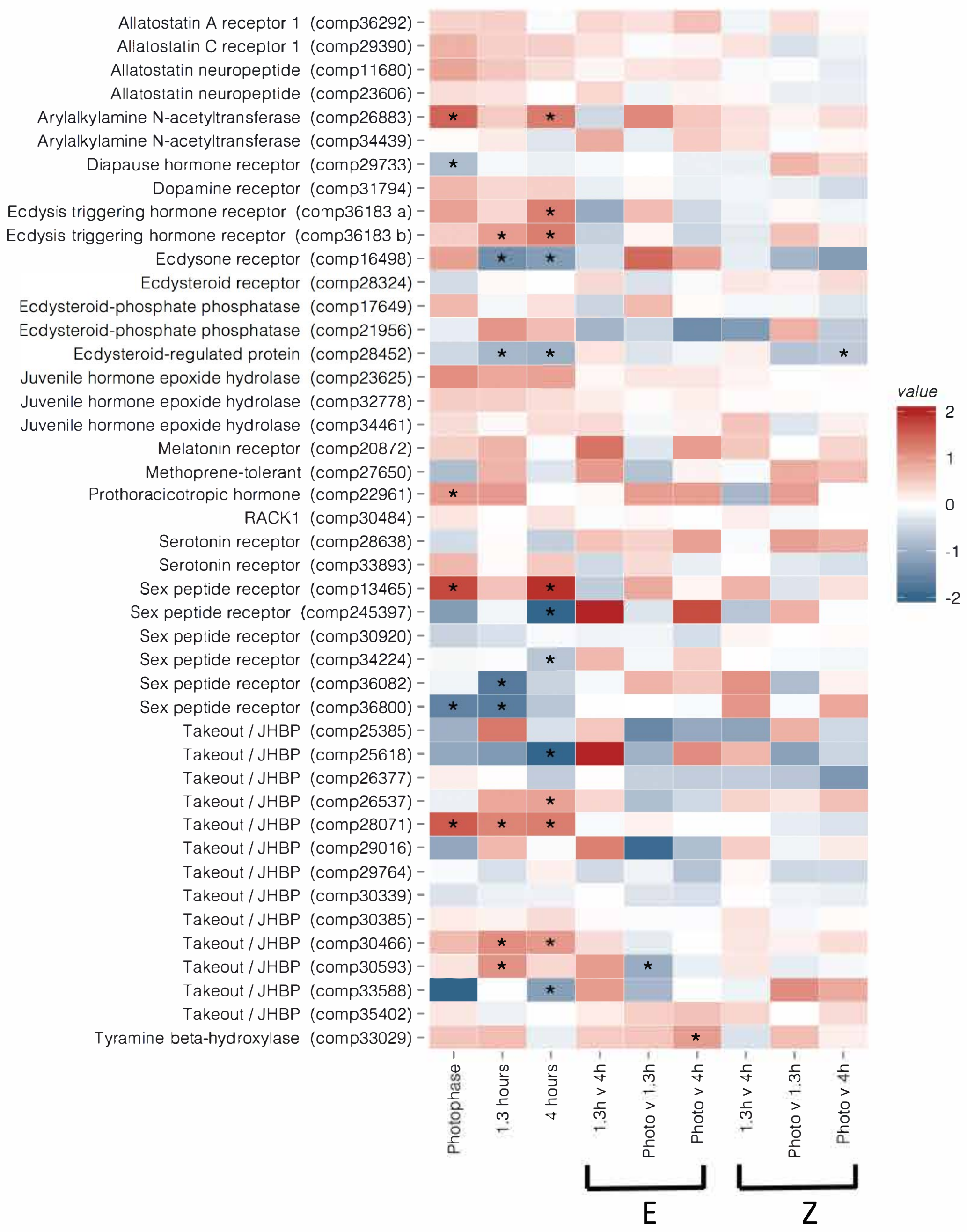

Figure S1.

Heatmap of relative expression of downstream candidate transcripts in each contrast. Photophase, 1.3 hour, and 4 hour contrasts are E vs. Z. Values are z-scored logFC, with red color indicating upregulation in the first term of each contrast, and blue color indicating upregulation in the second term of each contrast. Z-scores were capped to a minimum of -2 and a maximum of 2 for visualization. Asterisks indicate genes that are significantly differentially expressed (FDR-corrected p-value < 0.05) for a given contrast.

Table S1. Summary statistics of transcriptome assembly in Trinity.

Summary Statistics	
Unique Reads	61.5 M
Transcripts	78,236
Components	40,811
Minimum Read Length	201
Maximum Read Length	17,430
Mean Read Length	1,136
Median Read Length	691
N50	1,918

Supplemental Table 2. Overlapping differentially expressed genes between daily and seasonal time courses.

Candidate	Levy Comp	Wadsworth Comp	Gene Symbol	DE Diapause	DE Daily	Gene Name	Function
PDD Interval	comp9904_c0_seq1	comp34828_c0_seq1	CG30427	D1	Hr4	Fatty acyl-CoA reductase	Determination of lifespan
PDD Interval	comp48611_c0_seq1	comp19186_c0_seq1	CBG16133	D1	P,Hr1.3,Hr4	NA	NA
PDD Interval	comp36669_c0_seq10	comp25161_c0_seq1	*Magu*	D1	Hr4	NA	Regulation of life span, BMP signalling
PDD Interval	comp35947_c0_seq1	comp9449_c0_seq1	*GAPsec*	D1	P,Hr1.3,Hr4	GTPase-activating, SECIS-dependent read-through	Translation
PDD Interval	comp27771_c0_seq2	comp19707_c0_seq1	*CG10338*	D7	Hr1.3,Hr4	NA	NA
PDD Interval	comp26528_c0_seq1	comp18687_c0_seq1	*Plod*	D7	P,Hr1.3,Hr4	Procollagen lysyl hydroxylase	Collagen IV biosynthesis
PDD Interval	comp21518_c0_seq1	comp117543_c0_seq1	*Ttc39b*	D7	P	Tetratricopeptide repeat protein 39B	NA
PDD Interval	comp32386_c0_seq3	comp78006_c0_seq1	*Sca*	D7	P,Hr4	Scabrous	Neural patterning and interacts with the Notch pathway
PDD Interval	comp31831_c0_seq2	comp9302_c0_seq1	*Rngo*	D7	Hr1.3	Rings lost	Development
Hormonal	comp26537_c0_seq1	comp33893_c0_seq1	*to*	D1	Hr4	Takeout	Juvenile hormone binding protein
Hormonal	comp30466_c0_seq1	comp28293_c0_seq1	*to*	D1	Hr1.3,Hr4	Takeout	Juvenile hormone binding protein
Hormonal	comp30593_c0_seq1	comp22834_c0_seq1	*to*	D1,D7	Hr1.3	Takeout	Juvenile hormone binding protein
Hormonal	comp33588_c0_seq1	comp188240_c0_seq1	*to*	D7	P,Hr4	Takeout	Juvenile hormone binding protein
Hormonal	comp36183_c1_seq1	comp26395_c0_seq1	*ETHR*	D7	Hr1.3,Hr4	ecdysis triggering hormone receptor	Ecdysone binding

Table S3. Summary of GO term enrichment among significantly upregulated genes within each strain at each time point.

**E Strain**	**Process**	p-value	**Function**	p-value	**Component**	p-value
**Photophase**	**0**		**1**		**0**	
			**Hydrolase activity**	**0.00062**		
**Hour1**	**2**		**5**		**0**	
	light absorption	0.000846	cation: chloride symporter activity	0.0000954		
	reactive nitrogen species metabolic process	0.000846	anion transmembrane tranporter activity	0.000215		
			inorganic anion transmembrane tranporter activity	0.000271		
			chloride transmembrane transporter activity	0.000965		
			secondary active transmembrane transporter activity	0.000983		
**Hour4**	**5**		**0**		**0**	
	pigmentation	0.000538				
	developmental pigmentation	0.000538				
	phenol-containing compound metabolic process	0.000545				
	cuticle pigmentation	0.000697				
	regulation of melanization defense response	0.000903				

**Z Strain**	**Process**	p-value	**Function**	p-value	**Component**	p-value
**Photophase**	**6**		**2**		**0**	
	positive regulation of wound healing	0.000183	catalytic activity	0.000871		
	cell projection assembly	0.000298	endopeptidase inhibitor activity	0.000883		
	regulation of wound healing	0.000319				
	positive regulation of response to wounding	0.000515				
	lamellipodium assembly	0.00068				
	lamellipodium organization	0.00068				
**Hour1**	**5**		**3**		**2**	
	nucleobase biosynthetic process	0.000184	structural constituent of chitin-based cuticle	0.0000721	extracellular matrix	0.000253
	DNA metabolic process	0.000443	structural constituent of cuticle	0.000124	lipoamide complex	0.000559
	de novo pyrimidine nucleobase biosynthetic process	0.000671	2-methylpropanoyl transfering activity	0.000559		
	pyrimidine nucleobase biosynthetic process	0.000671				
	nucleobase metabolic process	0.000891				
**Hour4**	**0**		**1**		**0**	
			carboxylic ester hydrolase activity	0.000608		
